# Supplementary figures and images for: BH4 activates CaMKK2 and rescues the cardiomyopathic phenotype in rodent models of diabetes
Source: Life Sci Alliance. 2020 Jul 22;3(9):e201900619. doi: 10.26508/lsa.201900619 (PMC7383063; doi:10.26508/lsa.201900619)

Fig 4D. Western blot

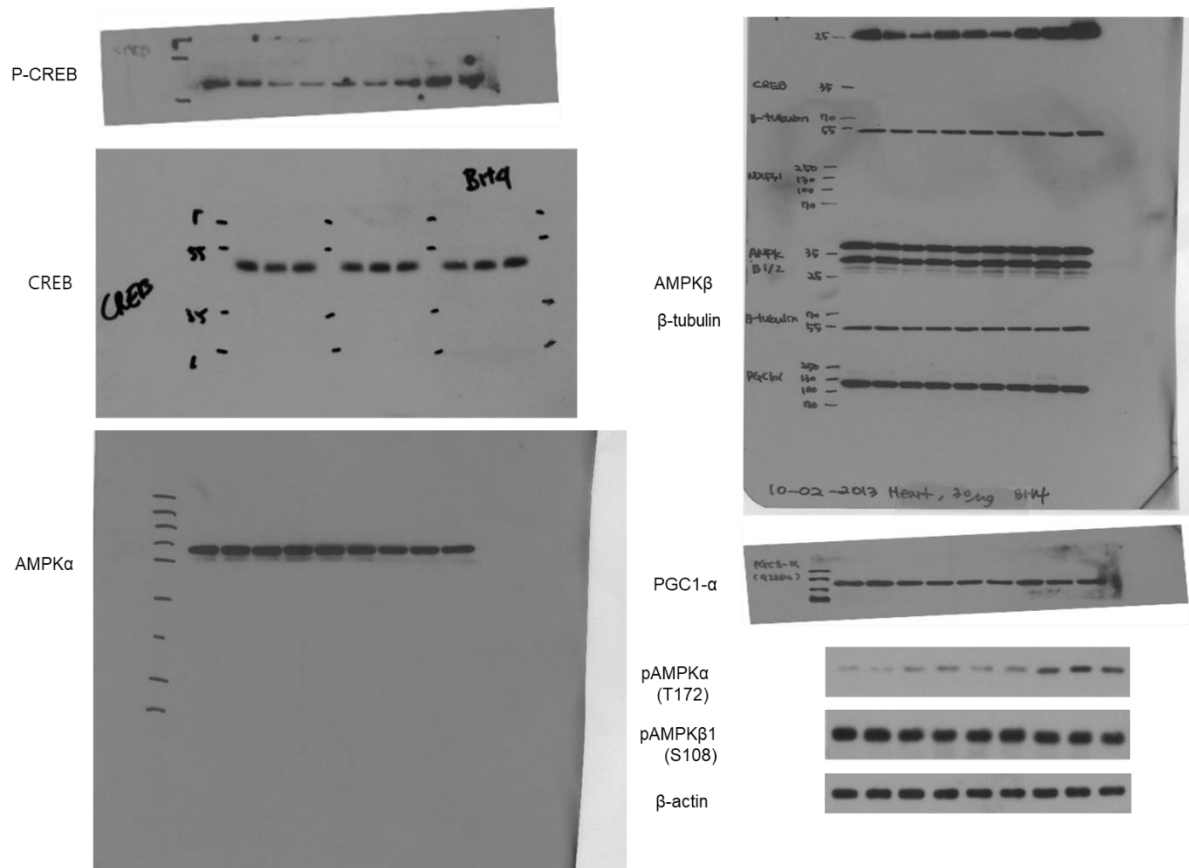

Fig 4E. WB

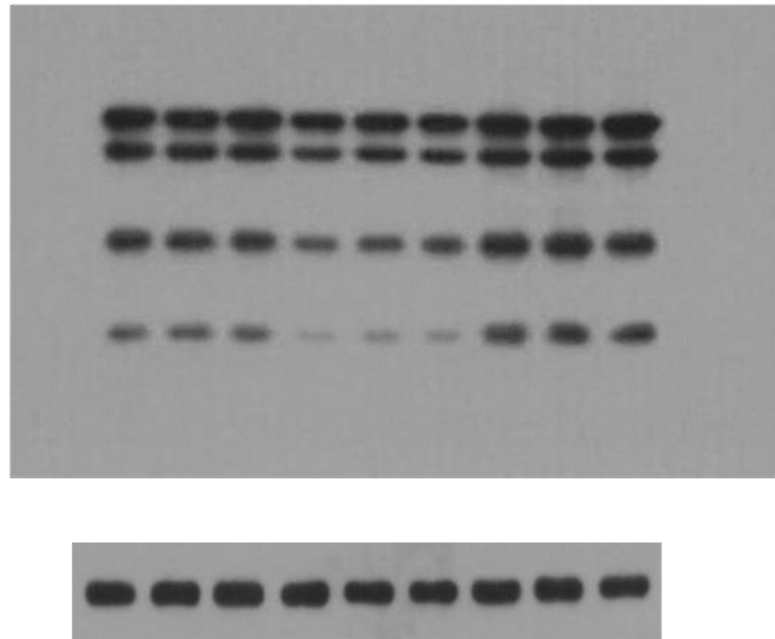

Supplement: Supplementary file 1 [file LSA-2019-00619_SdataF4.pdf]

Fig 6A. WB

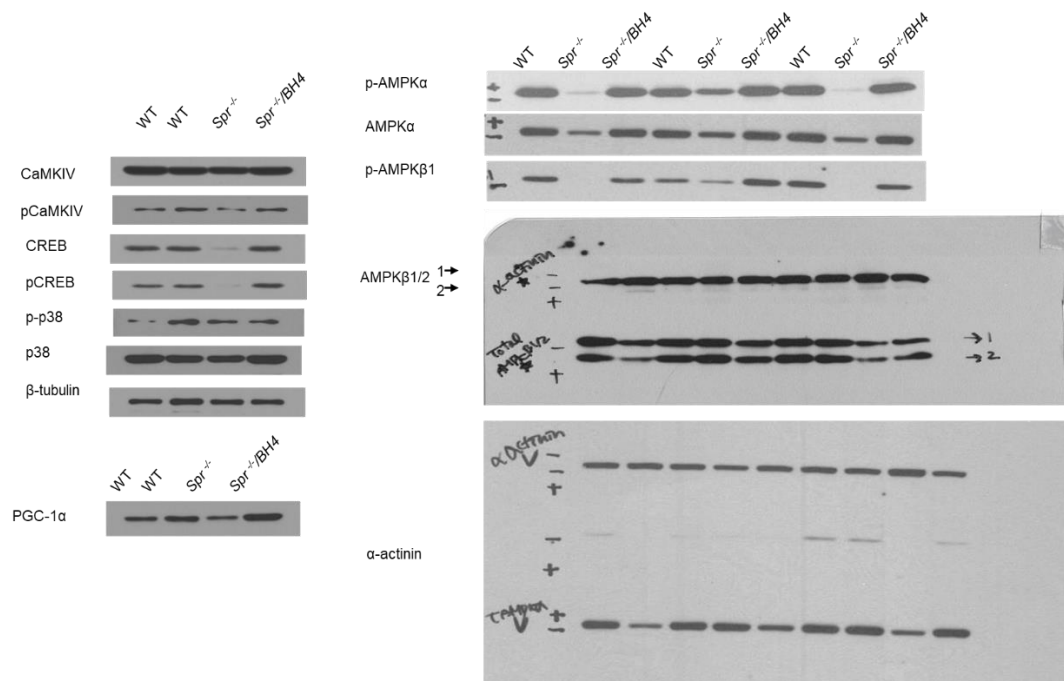

Supplement: Supplementary file 2 [file LSA-2019-00619_SdataF6.pdf]

Fig 7D. WB

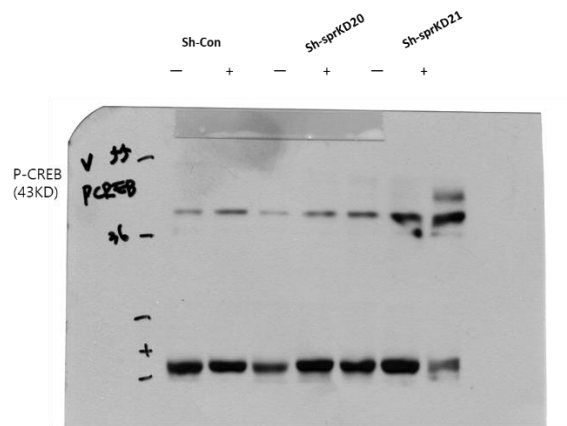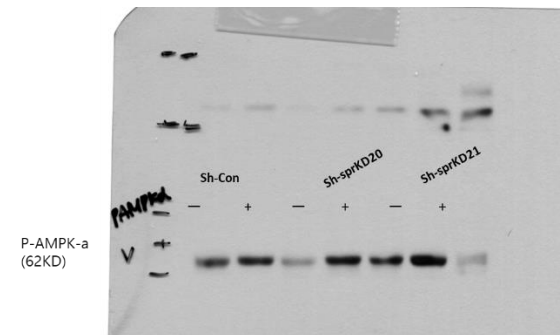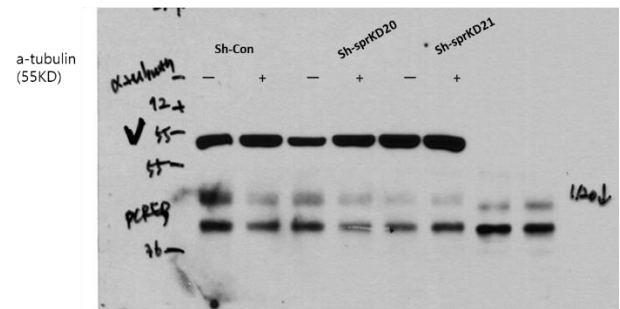

Fig 7E. WB

Original image

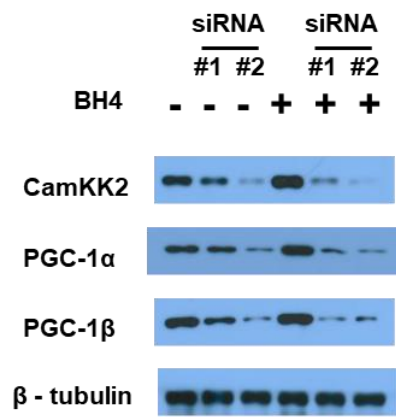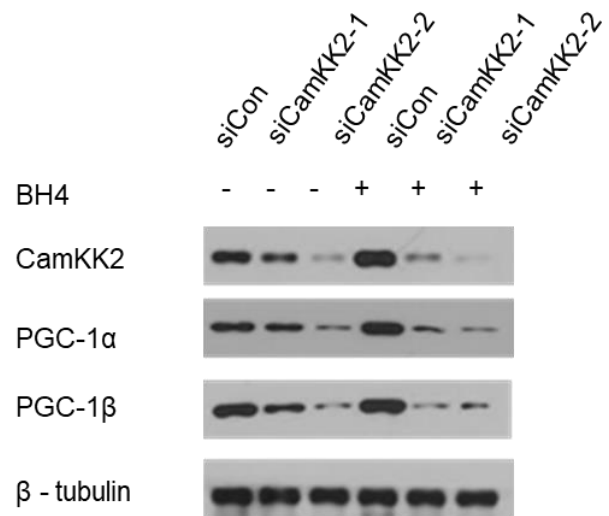

Supplement: Supplementary file 3 [file LSA-2019-00619_SdataF7.pdf]
