# Supplementary material for: BH4 activates CaMKK2 and rescues the cardiomyopathic phenotype in rodent models of diabetes
Source: Life Sci Alliance. 2020 Jul 22;3(9):e201900619. doi: 10.26508/lsa.201900619 (PMC7383063; doi:10.26508/lsa.201900619)
Supplement: Supplementary file 4 [file LSA-2019-00619_Supplemental_Data_1.docx]

**Supplementary Materials**

**1. Supplemental Experimental Procedures**

**Cardiac tissue histology analysis**

To assess changes in cardiac tissue histology, hematoxylin and eosin (H&E) staining was performed on formalin-fixed cardiac tissue from experimental animals. Isolated heart tissue was fixed in 10% polyformaldehyde, embedded in paraffin wax, and then cut into 4 μm sections that were subsequently stained with H&E. Stained sections were then characterized morphologically under an optical microscope at 500 × magnification.

**Zymography**

Zymography using gelatin containing polyacrylamide gel for detecting MMP activity was undertaken. MMP activity was indicated by clear zones on a blue background. The gels were scanned into a computer database and analysed with the NIH image.

**Measurement of TBARS**

The analysis was done using a previously published method([Hayashi et al, 2003](#_ENREF_1)). In brief, 50 μl plasma was mixed with 3 μl of 0.1 M 2,6-di-tert-butyl p-hydroxy-toluene in ethanol, 50 μl of 0.2 M phosphoric acid, and 6 μl of 0.1 M thiobarbituric acid. The mixture was then incubated at 90°C for 45 minutes. The samples were cooled on ice, after which 125 μl butanol and 10 μl saturated NaCl solution were added and the mixture was vortexed for 60 seconds. After centrifugation (one minute, 12 000 ×g), 62.5 μl of the supernatant fraction was measured in a fluorometer (λ530 nm excitation, λ590 nm emission).

**Measurement of cardiac BH_4_ concentration**

Cardiac tissues and isolated mitochondria were homogenized in 5 μl/mg of extraction buffer containing 50 mM Tris-HCl (pH 7.5), 1 mM EDTA, 0.1M KCl, 1 mM DTT, and 0.2 mM phenylmethylsulfonyl fluoride. Homogenates were centrifuged at 12,000*g* for 20 min at 4°C. The supernatants were subjected to differential oxidation in acidic (0.4 mol/l trichloroacetic acid) or alkaline (0.4 mol/l NaOH) solutions containing 1% I2/2% KI in 0.2M TCA or 1% I2/2% KI in 0.2M NaOH for 1 h in the dark. After centrifugation, 20 µl supernatant was injected into an HPLC system with a fluorescence detector. Excitation and emission wavelengths of 350 and 450 nm, respectively, were used to detect fluorescent BH_4_ and its oxidized species.

**Measurement of cardiac *Spr* activity**

The reaction was performed in phosphate buffer (0.1 mol/l, pH 7.5) containing a final concentration of 0.1 mmol/l sepiapterin, 0.2 mmol/l NADPH, and 5 µg cardiac tissue lysate. The enzymatic reaction was conducted at 37°C for 2 h in the dark, and the biopterin conversion rate was used as an index of *Spr* activity. The reaction was stopped by incubating with 10 µl iodine solution containing 2% K/1% I2 in 1 M HCL for 10 min in the dark. The precipitated proteins were removed by centrifugation at 13,000*g* for 10 min at room temperature. Excess iodine was then quenched by adding 25 µl 2% ascorbic acid. The resulting biopterin content was measured with an HPLC system (Waters Spherisorb 5 ODS-1 column, Gilson 321 model). Fluorescence was monitored with a HP 1046 fluorescent detector at 350 nm excitation and 450 nm emission wavelengths. The flow rate was maintained at 1.2 ml/min. The chromatographic profile was analyzed with the aid of EZstart chromatography software (Shimadzu).

**Electron microscopy analysis in *in situ* cardiac mitochondria**

The dissected heart fragments (1 mm^3^) from experimental animals were fixed in 2.5% glutaraldehyde in PBS solution at 4°C overnight, and then with 1% osmium tetroxide in PBS for 2 h. The tissues were washed, dehydrated, and embedded, and then semi-thin sections were cut (0.5–1 µm). Further ultra-sectioning (60–90 nm) was performed and then the slices were double stained with uranyl acetate and lead citrate and imaged using a JEM 1200 EX2 electron microscope (Jeol, Japan). Developed images were scanned on a flatbed scanner (Umax PowerLook 1100; Fremont, CA, USA) and analyzed using Image J software (http://rsb.info.nih.gov/ij/, 1.39u, NIH, USA).

**Isolation of single cardiac myocytes**

The heart was rapidly removed and retrogradely perfused through the aorta on a Langendorff system using oxygenated normal Tyrode’s solution containing (mM) 143 NaCl, 5.4 KCl, 1.8 CaCl_2_, 0.5 MgCl_2_, 5.5 glucose, and 5 HEPES (pH 7.4) adjusted with KOH at 37°C for 5–6 min to remove blood. Subsequently, hearts were perfused with Ca^2+^-free Tyrode’s solution for 5 min and then with Ca^2+^-free Tyrode’s solution containing 0.01% collagenase (1 mg/10 ml, Yakult) for 10–15 min and finally with Kraft-Brühe (KB) solution containing (mM) the following: 25 KCl, 10 KH_2_PO_4_, 16 KOH, 80 glutamic acid, 10 taurine, 14 oxalic acid, 10 HEPES, and 11 glucose, adjusted with KOH to pH 7.4 for 5–10 min for stabilization. Following the enzymatic digestion procedure, the left ventricle was excised and agitated mechanically in KB solution to obtain single myocytes.

**One-dimensional LC-ESI-MS/MS proteome analysis**

Coomassie blue-stained soluble cardiac mitochondria protein was separated on a 12% polyacrylamide gel by sodium dodecyl sulfate polyacrylamide gel electrophoresis (SDS–PAGE) ([Park et al, 2008](#_ENREF_3)). After SDS–PAGE, gels were washed three times with ddH_2_O for 5 min each, and then stained with Bio-Safe Coomassie Stain solution (Coomassie G250 Stain; Bio-Rad) for 1 h with gentle shaking at room temperature. Gels were destained by incubation in ddH_2_O for 30 min and washed three times in ddH_2_O for 10 min each ([Park et al, 2008](#_ENREF_3)).

In-gel tryptic digestion protein bands were excised from the Coomassie-stained gels and destained by incubating in 75 mM ammonium bicarbonate/40% Ethanol (1:1). Disulfides were reduced by treatment with 5 mM DTT/25 mM ammonium bicarbonate at 60°C for 30 min followed by alkylation with 55 mM iodoacetoamide at room temperature for 30 min. Gel pieces were then dehydrated in 100% ACN and dried. The gel pieces were swollen in 10 µl 25 mM ammonium bicarbonate buffer containing 20 µg/ml modified sequencing grade trypsin (Roche Applied Science) and incubated overnight at 37°C. The tryptic peptide mixture was eluted from the gel with 0.1% formic acid ([Park et al, 2008](#_ENREF_3)).

LC-ESI-MS/MS analysis LC-MS/MS analysis was performed using a ThermoFinnigan ProteomeX workstation LTQ linear ion trap MS (Thermo Electron, San Jose, CA, USA) equipped with NSI sources (Thermo Electron , San Jose, CA). Briefly, 12 μl of peptide sample from the in-gel digestion was injected and loaded onto a peptide trap cartridge (Agilent, Palo Alto, CA). Trapped peptides were eluted onto a 10 cm reversed-phase (RP) PicoFrit column packed in-house with 5 μm 300 Å pore size C18, then separated by gradient elution. The mobile phases consisted of H_2_O and ACN, and both contained 0.1% v/v formic acid. The flow rate was maintained at 200 nL/min. The gradient started at 2% ACN, reached 60% ACN in 50 min, 80% ACN in the next 5 min, and 100% H_2_O in the final 15 min. Data-dependent acquisition (m/z 400–1800) was enabled, and each MS survey scan was followed by five MS/MS scans within 30 s, with the dynamic exclusion option enabled. The spray voltage was 1.9 kV and the temperature of the ion transfer tube was set at 195°C. The normalized collision energy was set at 35% ([Park et al, 2008](#_ENREF_3)).

Data analysed tandem mass spectra were extracted and the charge state deconvoluted and de-isotoped using the Sorcerer 3.4 beta2 platform (Sorcerer software 3.1.4, Sorcerer Web interface 2.2.0 r334 and Trans-, Proteomic Pipeline 2.9.5). All MS/MS samples were analyzed using SEQUEST (ThermoFinnigan, San Jose, CA, USA; version v.27, rev. 11). SEQUEST was set to search the ipiRat 3.29 database (IPI ver.3.29, 40131 entries) with semitrypsin as the digestion enzyme. SEQUEST searched using a fragment ion mass tolerance of 1.00 Da and a parent ion tolerance of 1.5 Da. Iodoacetamide derivatized cysteine was specified as a fixed modification. Oxidation of methionine, iodoacetamide derivatized cysteine, and the phosphorylation of serine, threonine, and tyrosine were specified as variable modifications ([Park et al, 2008](#_ENREF_3)).

Protein identification Scaffold (version Scaffold-01_07_00, Proteome Software Inc., Portland, OR) was used to validate MS/MS-based peptide and protein identifications. Peptide identifications were accepted if they could be established at greater than 95.0% probability as specified by the Peptide Prophet algorithm and contained at least one identified peptide. Protein probabilities were assigned by the Protein Prophet algorithm. Proteins that contained similar peptides that could not be differentiated based on MS/MS analysis alone were grouped to satisfy the principles of parsimony. After identifying the proteins, each data set was used in a subtractive analysis by ProtAn X, an in-house analytic program ([Park et al, 2008](#_ENREF_3)).

**Western Blot Analysis**

Protein level of selected proteins, including CaMKIV, p-CaMKIV, CREB, p-CREB, p38MAPK, p-p38MAPK, AMPKα, β, p-AMPK α,β, α and β tubulin (Abcam, Cambridge, UK), and mitochondria transcription factor A, (*mt*TFA, Santa Cruz, CA, USA),were confirmed by Western blotting analysis. Briefly, the protein concentration of each sample was determined using the BCA protein assay. Samples containing 20 µg total proteins were subjected to 12% SDS-PAGE and then electrophoretically transferred to a polyvinylidene difluoride membrane. After the membrane was blocked with 5% non-fat dried milk in Tris-buffered saline/Tween-20 (TBST) for 1 h at room temperature, it was incubated for 2 h with randomly selected primary antibodies. After three washes in TBST, the membrane was incubated for 1 h with a specific peroxidase-coupled secondary antibody (Santa Cruz Biotechnology). The membrane was washed again, and the signals were visualized using an ECL image detector (LAS-3000; Fujifilm, Miyagi, Japan). Relative protein band intensities were analyzed by densitometry on a gel documentation system using Multi Gauge software (Fujifilm). Relative protein expression levels were normalized by intensities of β-tubulin, β-actin or GAPDH of each sample.

**Measurement of mitochondria membrane potential (ΔΨm)**

To monitor ΔΨm, isolated ventricular cardiomyocytes were washed in HEPES buffer (mmol/l: NaCl 130, KCl 4.7, MgSO4 1.2, KH2PO4 1.2, HEPES 10, Glucose 11 and CaCl2 0.2, pH 7.4) and then stained with 200 nM of the cationic dye tetramethylrhodamine ethyl ester perchlorate (TMRE) for 20 min at 37°C. Cells were then washed twice by centrifugation. TMRE-loaded cells were analyzed using FACScalibur (3000 cells/sample) (Becton Dickinson). After a 5 min period to attain a stable-state, a mitochondrial protonophore, FCCP was added (1 μM final concentration) and ΔΨm depolarization was monitored. TMRE fluorescence intensity was monitored at 582 nm (FL-2 channel). FACS data were analyzed using Cell Quest Pro version 5.2.1(Becton Dickinson)([Mattiasson, 2004](#_ENREF_2)).

**Measurement of mitochondrial and cellular oxygen consumption**

*Mitochondrial Oxygen Consumption*

Mitochondrial oxygen consumption was determined polarographically using a fiber optic oxygen monitor (Instech, Plymouth Meeting, PA, USA) with a 600 μl chamber at room temperature. Mitochondrial oxygen consumption was measured in an air-saturated (220 nmol O2/ml) respiration medium consisting of 250 mM sucrose, 0.1 mM EGTA, 2 mM phosphate buffer, 10 mM Tris–HCl, and 5 mM MgCl2 (pH 7.4), and added to a 1mg of permeabilized left ventricle tissue or a final concentration of 1.0 mg/ml of mitochondria. Respiratory rate of state 4 was determined in the presence of 5 mM malate and 5 mM glutamate or 5mM succinate with rotenone as respiratory substrates for complex I or complex II, respectively. State 3 (active) respiration rate was determined in the presence of 0.1 mM ADP. Oxygen uptake is expressed as ng-at O/min mg protein. Data were acquired at a sample rate of 100 ms and analyzed using the OOIsensors program (Ocean Optic Inc, FL, USA). Respiratory control ratio (RCR) was calculated by the ratio of state 3/state 4 oxygen consumption rate.

*Cellular Oxygen Consumption*

Cultured HL-1 cells were harvested and washed twice with PBS and then resuspended in fresh complete RPMI medium at a density of 2.5 × 10^7^ cells/mL. Each experiment measured oxygen consumption by 400 μL of resuspended cells in a 600-µL air-saturated chamber surrounded by a water-filled chamber maintained at 37 °C, using fiber-optic oxygen electrode (Instech). The oxygen concentration in fully oxygenated medium and cell cultures was taken to be 200 nmol/mL (200 μM). Oxygen consumption rates were calculated in nmoles per million cells per second.

**Detailed materials and method for complexes assay**

Complex I, II, III, and IV activities were measured in frozen isolated mitochondrial hearts using a 96-well plate–based assay. The activities of Complex I, II and IV were determined using the Complex I, II and IV Enzyme Activity Microplate Assay Kit (MitoSciences) on the basis of recommended protocol and settings described by the manufacturer. We used a modified MitoTOX^TM^ OXPHOS complex III activity kit (MitoSciences) to quantify Complex III activity. Data are presented as mOD/min.

We used 20 μg of mitochondrial extracts from each mouse heart for complex I activity assay (NADH dehydrogenase). The enzyme is immunocaptured within the wells of the microplate and activity is determined by following the oxidation of NADH to NAD+ and the simultaneous reduction of a dye which leads to increased absorbance at 450 nm.

Complex II (succinate-coenzyme Q reductase) enzyme was immunocaptured in wells coated with anti-ComplexII monoclonal antibody. After this in-well purification the production of ubiquinol by the enzyme is coupled to the reduction of the dye 2,6-diclorophenolindophenol (DCPIP) and a decreases in its absorbance at 600 nm.

For the complex III activity assay, we used 5mg/mL of mouse heart mitochondrial sample to replace bovine heart mitochondria provided in the kit without any additional drug treatment. The enzyme activity of complex III is measured in sample by monitoring the conversion of cytochrome c in its oxidize form to its reduced form, as a linear increase in absorbance at 550. Rotenone and KCN are used to inhibit complex I and IV, respectively.

Citrate synthase activity in frozen isolated mitochondria was measured using the citrate synthase assay kit (Sigma-Aldrich) and was carried out according to instructions from the manufacturer. The reaction mixture contained 30mM acetyl coenzyme A (acetyl-CoA), 10mM 5-dithiobis-2-nitrobenzoic acid (DTNB), and 20-40 µg mitochondrial protein and was initiated with 10 mM oxaloacetic acid (OAA) and monitored at 412 nm at 30s interval for 3 min at 25°C. Data are presented as units (µmole/ml/min).

**Tissue ATP concentrations**

Isolated hearts were homogenized in 2.5% (v/v) trichloroacetic acid (500 μl/20 mg tissue sample). After centrifugation, 400 μl of supernatant was neutralized with 1M Tris (80 μl). ATP concentration in the supernatant was determined using luciferin–luciferase luminometry and the ATP bioluminescent assay kit (catalog no. FL-AA, Sigma). Soluble protein concentration in the sample was measured with the bicinchoninic acid (BCA) assay according to the manufacturer’s instructions (Pierce BCA Protein Assay Kit, ThermoScientific). ATP concentrations are expressed as nanomoles of ATP per mg of tissue protein.

**Echocardiography measurement**

Echocardiographic measurement with a high resolution echocardiograph system Vivid 7(GE Healthcare) was used to detect cardiac structure alterations and cardiac function in vivo by using echocardiogram with a i13L-15MHz probe. We obtained M-mode IVS (intraventrcular septum), LVID (left ventricular interal diameter), LVPW (left ventricular posterior wall), EDV (end diastolic volume), ESV (end systolic volume), SV (stroke volume), EF (ejection fraction) and FS (fractional shortening) in conscious experimental animals. All data and images were saved and analyzed by an EchoPAC PC (GE Healthcare)

**In vitro SPR gene knock down study**

Lentiviral transduction particle containing shRNA of mouse SPR gene was purchased from Sigma Aldrich (SHCLNV-NM_011467). HL-1 cells, cardiomyocyte cell line, were kindly provided by Dr. William Claycomb (Louisiana State University Health Science Center, New Orleans, LA) (43). HL-1 cells were cultured as described before(44). For generation of stably knocked-down of SPR gene cell lines, HL-1 cells, (2 x 10^5^ cells in 6 well plate) were infected with shRNA lentiviral transduction particles and hexadimethrine bromide to a final concentration of 8 ug/ml, and then the cells were incubated for 4 hours and removed the medium containing lentiviral particles. After 24 hours, the infected cells were selected by culture in the presence of puromycin (1ug/ml). The selected clones were maintained in medium containing puromycin (0.1 ug/ml).

**In vitro Camkk2 gene knockdown study**

Two kinds of small interfering RNA (siRNA) duplexes siCamkk2-1 (Cat no. SI009940527; Qiagen, USA) and siCamkk2-2 (Cat no. SI009940534; Qiagen, USA) that target mice Camkk2 were synthesized. Optimum media (GIBCO, Carlsbad, CA, USA) and Lipofectamine RNAiMAX Reagent (Invitrogen, Carlsbad, CA, USA) were used for siRNA transfection into HL-1 cell. The siRNA was transfected into HL-1 cells with Lipofectamine RNAiMAX Reagent. After 48hr transfection, the cells were subjected for each experiment.

**Surface plasmon resonance analyses**
The interactions between BH4 and its direct target kinases were studied by using dual channel surface plasmon resonance (SPR) instrument (SR7500DC, Reichert, Depew, NY). Target full length proteins, CaMKIV and CaMKK2 (abcam, Cambridge, UK) were immobilized to the sensor chip surface by free amine coupling with a mixture of 0.1 M 1-ethyl-3-(3-dimethylaminopropyl)carbodiimide hydrochloride and 0.05 M *N*-hydroxysuccinimide injection followed by quenching the remaining activated carboxyl groups with 1 M ethanolamine, pH 8.5. A second reference cell was treated similarly without proteins. BH4 was prepared in running buffer (PBS-T, 0.01%) and injected for 3 min (association time) at a flow rate of 30 μl/min followed by a dissociation phase of 3 min. Nonspecific background binding was subtracted from each sensogram using SPR_V4017 Data Acquisition and Alignment Program (Reichert, Depew,NY). Binding rates and constants were independent of flow rate over a wide range. Best-fit kinetic parameters were obtained by global fitting analysis using Scrubber2 (Biologic Software,Australia)

**References**

Hayashi T, Sohmiya K, Ukimura A, Endoh S, Mori T, Shimomura H, Okabe M, Terasaki F, Kitaura Y (2003) Angiotensin II receptor blockade prevents microangiopathy and preserves diastolic function in the diabetic rat heart. Heart 89**:** 1236-1242

Mattiasson G (2004) Analysis of mitochondrial generation and release of reactive oxygen species. Cytometry A 62**:** 89-96

Park HJ, Kim BG, Lee SJ, Heo SH, Kim JY, Kwon TH, Lee EB, Ryoo HM, Cho JY (2008) Proteomic profiling of endothelial cells in human lung cancer. J Proteome Res 7**:** 1138-1150
